# Higher education student engagement in learning activities: Clarifying concepts and introducing a short-scale

**DOI:** 10.1371/journal.pone.0340391

**Published:** 2026-02-19

**Authors:** Feliciano Veiga, Johnmarshall Reeve, Carlota M. Veiga, Zi Yang Wong, Isabel Martínez, Nuno Archer de Carvalho, Anabela Pereira

**Affiliations:** 1 Unidade de Investigação e Desenvolvimento em Educação e Formação, Universidade de Lisboa, Instituto de Educação, Portugal; 2 Institute for Positive Psychology and Education, Australian Catholic University, Australia; 3 Centro de Investigação e Estudos de Sociologia, Instituto Universitário de Lisboa, Portugal; 4 National Institute of Education, Psychology and Child & Human Development Academic, Singapore; 5 Departamento de Psicología Universidad de Castilla-La Mancha, Spain; 6 Unidade de Investigação e Desenvolvimento em Educação e Formação, Universidade de Lisboa, Instituto de Educação, Portugal; 7 Universidade de Évora, Portugal; National University of Sciences and Technology, PAKISTAN

## Abstract

Student engagement plays a vital role in higher education due to its significant influence on academic outcomes, such as academic achievement and course completion. However, the concept of student engagement is often ambiguously defined, with a lack of distinction between engagement in learning and involvement in the academic community. Although the current student engagement measures have contributed to advancing knowledge in the area, they are often too long and exclude agentic engagement, which is seen as a key dimension in higher education. This research aimed to (a) clarify the concept of student engagement and the conceptual problems of the existing scales and (b) develop and validate a short, robust scale for measuring student engagement in learning activities, including agentic engagement. We conducted three studies with Portuguese higher education students: Study 1 developed the Higher Education Student Engagement in Learning Activities – a Short Scale, using exploratory factor analysis to assess cognitive, affective, behavioral, and agentic dimensions. Study 2 and Study 3, with different samples, evaluated the reliability and validity of the developed scale through confirmatory factor analysis. The results highlight the importance of a four-dimensional conceptual approach to student engagement and provide a validated framework for its measurement. This short scale clarifies the distinction between engagement in learning and in the academic community, and introduces agentic engagement as a key dimension. It offers a valuable tool for assessing student engagement in higher education, with implications for enhancing academic practices and outcomes.

## Introduction

Higher education institutions face diverse and complex challenges, including accessibility, quality of education, well-being, and student mental health [1,2]. One of the biggest problems for higher education institutions is the increase in dropout rates and low academic performance [2]. Since student engagement (SE) has been considered a protective factor for students against these problems, it makes perfect sense for academicians to be interested in monitoring instruments for such issues. Addressing them is critical, and SE has emerged as a key factor in promoting academic outcomes and student success [3]. Educators and researchers highly value SE because it predicts important outcomes in higher education, such as academic success [4], personal skills [5], educational attainment [6], well-being [7,8], and mental health [9]. Its malleable nature allows it to be influenced by the academic environment, thus making it a promising target for intervention in higher education [10,11].

Despite its importance, the concept of SE is often ambiguously defined, with researchers struggling to distinguish it from related constructs such as motivation and social context [6,12]. This conceptual confusion extends to the measurement of SE, where little agreement exists on the dimensions that define it and the items needed to assess them [13,14]. Although psychometrically strong, many of the existing scales often contain items on both engagement in learning activities and engagement in the academic community, and they often fail to distinguish between these two constructs, even though evidence shows that students may be engaged in one and not the other [15]. Moreover, most scales also did not include items on agentic engagement (i.e., students’ active contribution to their own learning), despite its crucial role in higher education [16–18].

To address these gaps, the present research aims to (a) clarify the conceptual haziness surrounding SE and (b) develop and validate a short, robust scale for measuring student engagement in learning activities (SELA) that includes agentic engagement. Therefore, we carried out three studies: Study 1 developed the Higher Education Student Engagement in Learning Activities – a Short Scale (HESELA-SS), using exploratory factor analysis (EFA) to assess cognitive, affective, behavioral, and agentic dimensions. Study 2, with a different sample, evaluated the scale’s reliability and multiple forms of validity using confirmatory factor analysis (CFA). Study 3 further tested the four-factor model and confirmed its structure and validity with a new sample.

### Overcoming conceptual confusion

In the higher education literature, two overarching theoretical approaches—behavioral and psychological—guide most research for measuring SE. From a *behavioral perspective*, SE focuses on students’ behaviors and institutional practices [12,19]. This perspective views SE as participatory behaviors in educational practices closely linked to learning [12]. The National Survey of Student Engagement [NSSE; 3; Table A1] was developed from this definition and inspired similar measures [20], being the most widely used instrument to measure SE in higher education [21]. However, some scholars [20,22] criticize the NSSE questionnaire because it is based on an overly broad definition of SE and evaluates educational experiences that are not engaging. The behavioral perspective overlooks the emotional and cognitive aspects of students’ learning experience, which the psychological perspective addresses.

The *psychological perspective* views SE as “an internal psycho-social process that evolves and varies in intensity” [12]. This approach, with a three-dimensional model encompassing emotional, behavioral, and cognitive dimensions, is one of the most popular [23]. Many measures based on this perspective have been developed, such as the Higher Education Student Engagement Scale [14]. However, Kahu notes that the “key limitations of the psychological perspective center on a lack of definition and differentiation between the dimensions” [12]. In some scales within this approach, some items refer to antecedents or consequences of SE and are not engagement indicators [6,12,15,21]. A recent meta-analysis [22] highlighted the inconsistent operationalization of these dimensions in the field. Therefore, this was considered in our research, which is inserted in the psychological perspective of SE, going beyond observation and focusing on what students think, feel, and act as agents during learning activities.

### Focusing on student engagement in learning activities (SELA)

The confusion in the SE concept has reached a critical point [6,15], to the extent that it was characterized as “one of the most widely misused and overgeneralized constructs found in the educational and psychological sciences” [24]. Consider the case of overgeneralization. This refers to including numerous variables in the SE concept, encompassing any variable related to academic performance and retention [15,20]. For example, researchers have included university belonging [13], behavioral adequacy [9], and dropout rates [25] as indicators of the construct. This issue was also illustrated in a recent meta-analysis [22], which revealed that numerous indicators operationalized engagement dimensions inconsistently.

Part of the issue of overgeneralization is object ambiguity. It occurs when it is unclear what object the student is engaging with, which is problematic because the meaning of SE depends on the object of focus [15]. This is most evident in conceptualizations and measurement tools that ask students about their engagement in extracurricular activities [26], classes [27], campus life [13], university courses [28], interactions with colleagues and professors [14], or a combination of learning activities and the academic community [28,29].

**Fig 1:**
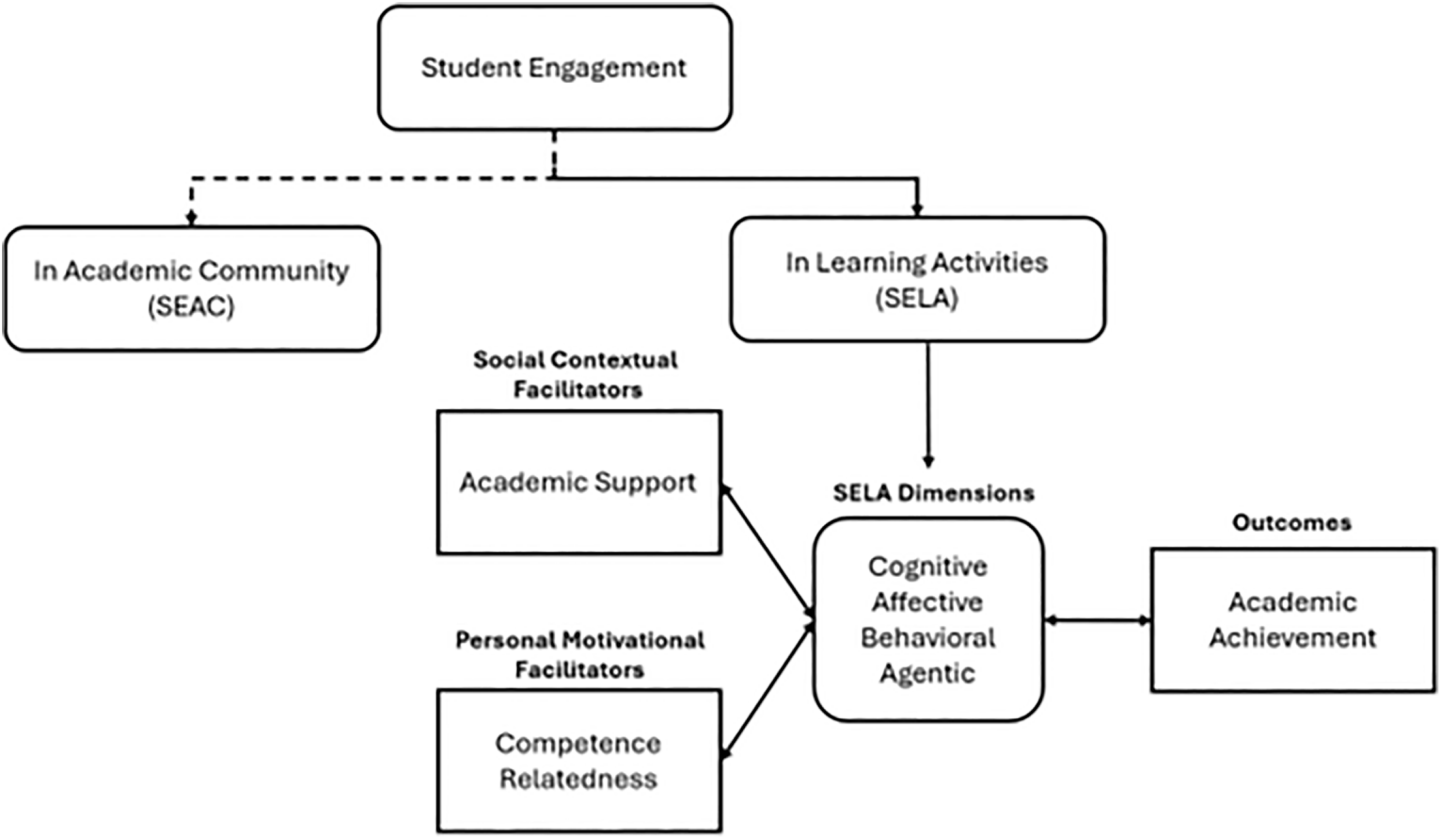
Dual components of se and four-dimensionality of SELA: conceptual framework.

To address these conceptual concerns, Wong and Liem [15] proposed the Dual Component Framework of Student Engagement (DCFSE). According to this framework, SE is a higher-order concept that encompasses two related but distinct constructs: (a) SELA and (b) student engagement in the academic community (SEAC). SELA is a multidimensional construct that captures how students think, feel, behave, and act as agents during learning activities. It is a malleable state that varies with the context, such as the support provided by the academy [8,30] and the characteristics of the learning activity. SELA is distinguished from contextual, personal, and outcome variables (Fig 1) [20,31].

Differently, SEAC refers to the student’s relational attachment to people in the academic community and their sense of belonging as a member of the university [13]. While SELA is a psychological process [15], SEAC is a sociological process [25]. Acknowledging the distinction between SELA and SEAC, this research focused on SELA.

### Adopting a four-dimensional perspective

SELA has been widely accepted as a multidimensional construct, but there is little agreement on the number or names of these dimensions [15,20,21]. Nevertheless, contemporary SE researchers agree that engagement consists of at least three dimensions: cognitive, behavioral, and affective [21]. Cognitive engagement refers to sophisticated learning strategies, such as elaboration and problem-solving, connecting new information with prior knowledge, and applying knowledge in different contexts [21,32]. Affective engagement is the positive emotional connection between the student and the learning activity, measured by interest, enthusiasm, and enjoyment in learning [7,20]. Behavioral engagement implies the student’s observable conduct during learning activities, including attention, participation, and persistence [23,25].

Beyond the tripartite conceptualization of SE, some researchers have called for the addition of a fourth dimension, namely agentic engagement. Agentic engagement implies the student’s initiative during learning activities, such as asking questions, expressing opinions, and making suggestions, positively contributing to learning [9,32]. Higher education has greatly emphasized the importance of creating conditions for active student participation in their learning process [2,16]. Despite this, current SE measures in higher education often do not include agentic engagement [18].

One argument for incorporating agentic engagement is based on its incremental validity. This dimension helps explain variances in learning outcomes that other types of engagement might not account for alone [32]. Additionally, studies have shown that agentic engagement positively correlates with important educational outcomes, such as academic performance, student autonomy, intrinsic motivation, critical thinking, resilience, and civic responsibility [9,10].

Furthermore, incorporating agentic engagement into SE scales adds a proactive quality to the otherwise reactive nature of the other engagement dimensions [17]. Therefore, adding this dimension can improve engagement measures’ overall reliability and validity [27]. The present research thus adopts a four-dimensional perspective that includes the agentic dimension (Fig 1).

### Higher education SELA measures

The existing SE scales have been instrumental in advancing understanding of student engagement, providing a solid foundation for subsequent research [19]. Reliable and valid SE instruments have significantly advanced knowledge in the field, allowing universities to monitor and identify areas where educational practices need improvement to enhance SE [20,21]. However, some instruments’ numerous dimensions and items can undermine useful analysis.

S1 Table provides a comparative overview of the most common scales used to measure SE in higher education. The National Survey of Student Engagement (NSSE) [3,4] has the highest number of items (100), while the University Student Engagement Inventory (USEI) [33] has the fewest (15 items). The USEI also has the fewest dimensions (three), whereas the Student Engagement Scale (StES) [26] has the most dimensions (nine). Notably, none of these scales include agentic engagement, suggesting a potential area for future development in SE measurement tools. All scales present adequate reliability and validity values, with the StES showing a lower value (α = .63).

To varying extents, all scales include SELA and SEAC items, which do not align with Wong and Liem’s DCFSE [15]. For instance, the Student Engagement Scale [26] includes contrasting items like “I set my own learning goals” and “I feel like I belong to the campus.” The Higher Education Student Engagement Scale [14] includes contrasting items such as “I spend a lot of time studying by myself” and “I feel a sense of belonging to the university community.” The USEI [33] includes both “I discuss matters that I learned in class with people outside the school” and “I do not feel very accomplished in this school.” The NSSE [3] includes items like “I actively participate in class discussions” and “I feel a sense of belonging to the university community.” As for the length, only the USEI approaches a short-scale measurement format. Scales can be considered “short” when they have a small number of items but still capture the main dimensions and indicators of the measured construct. The minimum number of items per dimension for a short scale can be as few as two, and the maximum generally includes up to four [34]. Short scales offer several advantages [34,35]. Firstly, they are time-efficient, allowing for quick administration and scoring. This efficiency often leads to higher response rates and reduced participant fatigue. Secondly, short scales are cost-effective, requiring fewer resources, making them accessible to researchers with limited budgets. Their simplicity in administration and interpretation minimizes errors, making them user-friendly and less daunting for participants [35]. Their flexibility allows for easy integration into more extensive assessments or use alongside other measures, enhancing their versatility across different settings and populations [34].

Considering (a) the perspectives of SE in higher education, (b) the lack of differentiation between SELA and SEAC among many of the existing higher education SE scales, (c) the lack of scales that measure agentic engagement in higher education contexts, and (d) the advantages of short scales and their scarcity in higher education, we developed the HESELA-SS from a four-dimensional psychological perspective that includes the agentic engagement [27,32].

## Method

### Participants

We conducted this study in three phases, each involving different student cohorts to ensure a broader and more representative sample. This approach allowed us to capture variations across academic backgrounds and institutional contexts while testing the consistency of the proposed model over time. By incorporating different groups of students in each phase, we aimed to enhance the generalizability of the findings.

Therefore, this research involved 996 higher-education Portuguese students, divided into three studies with different samples, using non-probabilistic convenience sampling. To mitigate any biases associated with this type of sampling and increase the sample’s representativeness [36], we randomly selected a subset of participants within each sample, ensuring that every student within the convenience sample had an equal chance of being selected. We recruited participants from a diverse range of Portuguese higher education institutions, including both public and private universities. These institutions represent various academic disciplines, ensuring a heterogeneous sample that reflects different educational contexts and student profiles. Furthermore, these academies served students from all socio-economic levels, mostly medium level. Students were from different geographic zones: north, center, and south. Most Portuguese universities are public and offer both undergraduate and postgraduate programs across a wide range of fields, including social sciences, arts, and engineering. Teaching is mainly face-to-face, with some hybrid or online components. Undergraduate class sizes typically range from 30 to 60 students. The average graduation rate in public institutions is approximately 70%.

In Study 1, 205 students participated (Table 1), with 81.5% identifying as female, 46.8% studying Psychology, and 76.6% of the participants were first-year students.

**Table 1:** Samples for Study 1, Study 2, and Study 3.

	Study 1 (*N* = 205)		Study 2 (*N* = 404)		Study 3 (*N* = 387)	
Variable	*n*	%	*n*	%	*n*	%
Gender						
Female	167	81.5	308	76.2	322	83.2
Male	36	17.6	88	21.8	58	15.0
Non-binary	2	1.0	8	2.0	7	1.8
Course						
Psychology	96	46.8	96	23.8	136	35.1
Basic Education	52	25.4	128	31.7	177	45.7
Social Education	57	27.8	180	44.6	0	0.0
Architecture	0	0.0	0	0.0	74	19.1
Academic Year						
1^st^	157	76.6	251	62.1	163	42.1
3^rd^	48	23.4	153	37.9	224	57.9

Study 2 involved a sample of 404 students, comprising 76.2% females, 44.5% were studying Basic Education, and most were first-year students (62.1%).

Study 3 included 387 higher education students, of whom 83.2% identified as female, 45.7% were studying Basic Education, and 42.1% were first-year students.

### Measures

#### HESELA-SS: items development.

We developed the items for the HESELA-SS based on Wong and Liem’s DCFSE and the four-dimensional nature of the SE construct. First, we conducted a literature review to identify scales assessing higher education SELA. This review included a variety of measures, such as scales that measure cognitive engagement [3,13], affective engagement [14], and behavioral engagement [26,33], as these are highly valued in research [20]. We also considered agentic engagement due to its incremental validity [9,32].

Second, we conducted semi-structured interviews with a heterogeneous group of 11 higher-education students to determine the need for additional items. To ensure the group’s heterogeneity, we selected the students considering their similar distribution by academic year (1st and 3rd), age, gender, course (education and psychology), and region (north, center, and south of the country). We informed students about the meaning of SE in its four dimensions and then asked to answer an open question about each one.

As a result, we initially developed 52 items, which we then presented to a heterogeneous group of eight experts in education and psychology in higher education. We followed the same criteria used for selecting the group of students to ensure the heterogeneity of the group of experts. We informed the experts about the meaning of engagement in its four dimensions, and their task was to classify each item into its most appropriate engagement dimension. We used the Kappa index to measure agreement between experts [37]. The item-dimension concordance was.79 for the cognitive dimension,.88 for the affective dimension,.82 for the behavioral dimension, and.85 for the agentic dimension, indicating good agreement [37].

Based on the consultation and feedback from these experts, we revised 10 of the 52 items and deleted 8. We administered the remaining 44 items to the pilot sample to evaluate each item’s clarity and understanding. This process resulted in 40 items, with 10 for each dimension (S2 Table), rated on a 5-point Likert scale, ranging from 1 (strongly disagree) to 5 (strongly agree). We used no reverse-scored items, and items were grouped by dimension. Although the HESELA-SS initially considered 40 items, our objective—based on literature highlighting the benefits of shorter scales [e.g., 34]—was to retain as few items as possible, while still capturing the core dimensions and indicators of the SELA construct.

### Indicators for concurrent validity

We assessed concurrent validity by correlating HESELA-SS scores with the “Student Engagement Questionnaire” [SEQ; 38], because the SEQ assesses SELA and demonstrates good internal reliability and construct validity across different cultures. We used the following items from Lam et al.’s [38] SEQ: items 3, 9, and 12 for the cognitive dimension (ω = .75); items 1, 4, and 6 for the affective dimension (ω = .72); and items 8, 9, and 12 for the behavioral dimension (ω = .76). The total score of these nine items also showed adequate internal consistency (ω = .77). Like HESELA-SS, participants answered this and the subsequent measures using a 5-point Likert scale (ranging from 1 = strongly disagree to 5 = strongly agree).

### Indicators for predictive validity

Regarding predictive validity, we used academic support by professors as a social-contextual indicator [31], perceived competence and perceived relatedness as personal motivational indicators [39], and academic achievement as an outcome. Academic support refers to the follow-up professors provide students, including academic guidance, feedback, and availability to help (ω = .92, ten items, e.g., “My professors help me understand how I need to improve”) [40]. Perceived competence refers to feeling capable and masterful while interacting with the environment (ω = .82, three items extracted from Deci et al. [39]; e.g., “I consider myself able to learn successfully”). Perceived relatedness refers to feelings of belonging and connection with others (ω = .80, three items from Deci et al. [39]; e.g., “I feel emotionally close to the people around me”). To assess academic achievement, students answered the question “Rate your academic achievement in the faculty or institute,” with answers on a 5-point Likert scale (from very low, 1, to very high, 5).

### Procedure

The Ethics Committee of the Institute of Education of the University of Lisbon, Portugal, approved the proposed research (N.º 1525 Proc. IDOK de 27/11/2023). Students’ participation was voluntary and without any incentive. We provided interested students with a website explaining the goals, the questionnaires, and the research procedures. We ensured the confidentiality and anonymity of the data collected by the written informed consent that was included in the beginning section of the questionnaires. Data collection took place during the 2023–2024 academic year, starting on November 27th, 2023, and ending on June 4th, 2024. Students completed the survey in a classroom through the Google Forms online platform under the guidance of a professor, and it took approximately 10 minutes. The data collection process comprised attention checks. We excluded participants who hurriedly completed the survey, as well as those who constantly responded in the same manner to a series of items and those who provided inconsistent responses to equivalent items. Since the online platform required an answer to all questions, there were no missing data.

### Data analyses

For the data analyses, we used IBM SPSS and AMOS, version 27. First, we screened the data for univariate outliers, z > ±3.29 [41], using the Mahalanobis distance and the critical value for each case based on the chi-square distribution values to assess the presence of multivariate outliers. We removed 7 outliers in Study 1, 19 from the dataset in the Study 2 sample, and 21 from the Study 3 sample. Initially, we examined the normality of the distribution for all variables across the three studies. The skewness and kurtosis values fell within acceptable limits: skewness < |3.0| and kurtosis < |8.0| [42]. Furthermore, to ensure the absence of multicollinearity, all tolerance values exceeded.10, and variance inflation factor (VIF) values were below 10 [42]. For all the analyses, we considered results statistically significant when the *p*-value was below.05 for a 95% confidence interval.

In Study 1, we assessed construct validity through an exploratory factor analysis (EFA) with varimax rotation, adhering to conventional practices in the SE literature [9,13]. The determination of the number of factors and retained items was based on several criteria: commonalities close to or exceeding.50, eigenvalues greater than 1, contribution to variance, scree plot analysis, parallel analysis, and theoretical considerations such as conceptual clarity and interpretability [41,43]. We classified factor loadings as excellent (>.71), very good (>.61), and good (>.55) [44], and a factor solution that accounted for sixty percent of the total variance as satisfactory [45].

In Studies 2 and 3, we conducted a four-factor confirmatory factor analysis (CFA) using maximum likelihood estimation considering model fit as satisfactory when the *χ*²/df ratio fell between 1 and 3 [46], with Comparative Fit Index (CFI) and Goodness of Fit Index (GFI) values exceeding.90 [42] and Root Mean Square Error of Approximation (RMSEA) values below.08 [45]. Furthermore, in Study 2, we tested a second-order model, anticipating that the four dimensions of engagement would align within a broader meta-construct.

We also examined standardized residuals, and values below |2.5| did not suggest issues with the model [45]. We assessed convergent validity by calculating the average variance extracted (AVE), with values of ≥.50 considered acceptable [47]. We verified discriminant validity by ensuring that the square root of the AVE was greater than the correlations among the scores of the four dimensions and that AVE values surpassed the maximum shared variance (MSV) values [45,47]. To evaluate reliability, we computed McDonald’s omega (*ω*) and Composite Reliability (CR), with values exceeding.70 deemed satisfactory [42]. We also analyzed reliability by test-retest in Study 2 by computing Pearson correlation coefficients.

Pearson correlation coefficients assessed concurrent validity, with the strength of the correlations evaluated based on Cohen’s [48] criteria: *r* = .10 representing a small effect, *r* = .30 indicating a moderate effect, and *r* = .50 signifying a large effect. We conducted multiple linear regressions to analyze predictive validity.

To investigate measurement invariance, we performed multigroup confirmatory factor analyses (CFAs) to test the configural, metric (weak), and scalar (strong) invariance of the four-factor model of self-efficacy (SE) based on gender (male vs female) and academic year (1st *vs* 3rd year). We established a baseline unconstrained model as a reference for subsequent analyses [49] and then constrained factor loadings and item intercepts to be equal across groups for testing metric and scalar invariance, respectively. We used two criteria to evaluate invariance at different levels: a change in CFI (ΔCFI) of less than.01 between the constrained and free models [50] and a Δ*χ*^*2*^ test indicating no statistically significant difference between the fit of the constrained and free models [51].

## Results in HESELA-SS

### Construct Validity

In Study 1, two EFAs used the 40 candidate items in the S2 Table to select the final 12 items. The first EFA identified 13 items to exclude based on small factor loadings and cross-loadings. In the second EFA, the scree plot and the parallel analysis involving the remaining 27 candidate items supported a four-factor solution, explaining 58.42% of the variance. Of these items, we selected the three best in each dimension. The resulting four-factor, 12-item scale shown in Table 2 explained 69.9% of the total variance of the measure. All factor loadings were excellent (>.75), except for the third item on the behavioral engagement factor (.65). The McDonald’s Omega reliability coefficients were acceptable:.69 (cognitive),.83 (affective),.72 (behavioral),.84 (agentic).

**Table 2:** Items of the HESELA-SS validated in the EFA (Study 1).

HESELA-SS	Agentic	Affective	Behavioral	Cognitive
	19.39%	18.87%	15.83%	15.80%
09 (10). I let the professors know what I think about the subjects to learn.	.89			
04 (11). During class, I ask questions about the content to learn.	.84			
03 (12). I give the professors suggestions to create innovative tasks.	.81			
02 (04). I like what I am learning in classes.		.85		
03 (05). I feel enthusiastic about what I am going to learn this academic year.		.84		
08 (06). I feel what we are learning in class is very interesting.		.81		
08 (07). I remain very attentive to what the professors teach.			.82	
01 (08). I pay attention in classes.			.81	
04 (09). I work as much as I can when we start a new subject.			.65	
03 (01). When I learn new subjects, I try to associate them with what I have learned in other classes.				.78
01 (02). When I study, I try to relate what I am learning with something I already know.				.76
09 (03). I try to integrate my previously learned knowledge to solve new problems.				.75

**Fig 2:**
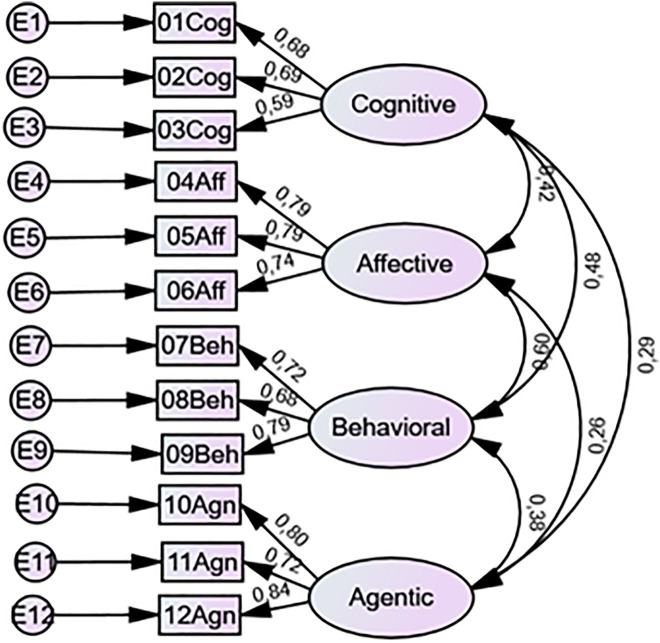
Items of the HESELA-SS Validated in the Confirmatory Factor Analysis: Four-factor Model (Study 2).

Only the three items with the highest loading by dimension are presented (Study 1). The number preceding each item refers to its original order on the HESELA-SS. The number in parentheses refers to the item number in the Study 2 CFA presented in Fig 2.

In Study 2, we examined this 12-item, four-dimensional structure using CFA and a new sample of participants. Fig 2 presents the results of the CFA, including factor loadings and factor correlations. We found a good model fit, χ^2^ (48) = 87.32, χ^2^/*df* = 1.82, GFI = .966; CFI = .976; RMSEA = .045. Factor loadings were mostly very good and excellent, all higher than.58 (all *p* < .001).

We examined a second-order model (S1 Fig), with SE as a higher-order factor and cognitive, affective, behavioral, and agentic engagement as first-order factors. The model showed a good model fit, χ^2^ (50) = 88.97, χ^2^/*df* = 1.78, GFI = .965; CFI = .977; RMSEA = .044. All four factors were significantly related to SE (*p* < .001). The two models fit the data equally well, with no significant difference, χ^2^ (2) = 1.65, *p* = .438. Therefore, reporting HESELA-SS scores by dimension or as a 12-item aggregate score is equally reasonable. We reviewed standardized residuals for each model, with all values remaining below |2.5|, following the recommendations [45].

In Study 3, this 12-item, four-dimensional structure also presented a good model fit, χ^2^ (48) = 103.84, χ^2^/*df* = 2.16, GFI = .958; CFI = .977; RMSEA = .055. Factor loadings were mostly very good and excellent, all higher than.54 (all *p* < .001). When we analyzed the standardized residuals, only two were between |2.5| and |4.0|; considering that the respective items did not show other problems, we made no changes to the models [45].

### Reliability

Table 3 presents the descriptive statistics and reliability regarding the four SE dimensions in Studies 2 and 3. The 3-item composite reliabilities (CR) ranged between.70 and.83 in Study 2 and between.80 and.88 in Study 3. We also analyzed reliability by test-retest. In Study 2, a group of participants (*N* = 92) completed the questionnaire twice, with a three-month interval. The correlations between the scale dimensions in the two administrations (ranging from *r* = .71 to *r* = .79) indicated satisfactory test-retest reliability.

**Table 3:** Reliability and descriptive statistics for the HESELA-SS (Studies 2 and 3).

	Mean	*SD*	Skewness	Kurtosis	CR	Test-retest
Study 2						
Cognitive	3.46	.65	−.02	.03	.70	.76^***^
Affective	3.90	.67	−.63	.77	.82	.78^***^
Behavioral	3.26	.74	−.24	.43	.78	.71^***^
Agentic	2.64	.91	−.02	−.43	.83	.79^***^
Total HESELA-SS	3.32	.52	−.17	1.02	---	.89^***^
Study 3						
Cognitive	4.25	.57	−.14	−.53	.88	—
Affective	3.83	.75	−.30	−.31	.87	—
Behavioral	3.90	.69	−.27	−.16	.84	—
Agentic	3.04	.86	.02	−.28	.80	—
Total HESELA-SS	3.75	.51	.17	−.25	.85	—

The range of scores for each item on the scale is 1–5; ^**^ *p* < .001

### Convergent and discriminant validity

Table 4 provides data on the convergent and discriminant validity for Studies 2 and 3. In Study 2, all four dimensions showed acceptable AVE values (>.50), except for the cognitive dimension (.43). However, since its CR was higher than.60 (see Table 2), we considered convergent validity adequate [47]. In Study 3, all AVE values are > .50, supporting convergent validity. Additionally, in both Studies, the square root of the AVE values was higher than the observed correlations between the dimensions, and AVE values were higher than MSV values, thus establishing discriminant validity [45].

**Table 4:** Convergent and discriminant validity for the HESELA-SS (Studies 2 and 3).

Dimensions	AVE	MSV	Agentic	Cognitive	Affective	Behavioral
Study 2						
Agentic	.62	.15	.**79**			
Cognitive	.43	.23	.29	**.66**		
Affective	.60	.36	.27	.43	**.78**	
Behavioral	.54	.36	.38	.48	.60	**.74**
Study 3						
Agentic	.58	.16	**.76**			
Cognitive	.71	.23	.27	**.85**		
Affective	.70	.23	.37	.48	**.84**	
Behavioral	.65	.22	.40	.47	.47	**.83**

All correlations are statistically significant at *p* < .001. AVE = Average variance extracted (convergent validity). MSV = Maximum shared squared variance (discriminant validity). The square root of AVE is presented in bold.

### Concurrent Validity

Table 5 shows a strong positive correlation between the total scores for the HESELA-SS and the SEQ (*r* = .71, *p* < .001) in Study 2. Each HESELA-SS dimension correlated positively and significantly with its corresponding SEQ dimension (*r* = .63 for behavioral, 61 for affective,.43 for cognitive, and.35 for agentic, all *p* < .001).

**Table 5:** Correlations between the HESELA-SS and the SEQ (Study 2).

		SEQ			
		Cognitive	Affective	Behavioral	Total
HESELA-SS	Cognitive	.44^***^	.27^***^	.28^***^	.43^***^
	Affective	.30^***^	.66^***^	.39^***^	.61^***^
	Behavioral	.30^***^	.52^***^	.57^***^	.63^***^
	Agentic	.18^**^	.29^***^	.30^***^	.35^***^
	Total	.42^***^	.61^***^	.55^***^	.71^***^

** *p* < .01; *** *p* < .001

### Predictive validity

All four HESELA-SS dimensions correlated positively and significantly with the three outcome criterion variables. After controlling for the variance explained by the other three dimensions, a multiple linear regression examined whether each dimension could predict independent variance in each criterion.

As shown in Table 6, all four four-predictor models were statistically significant in Study 2 (*p* < .001). For academic support, the individually significant dimensions were affective (*p* = .001), behavioral, and agentic (both *p* < .001). For competence, the individually significant dimensions were affective (*p* = .001), behavioral (*p* = .002), and agentic (*p* < .001). For relatedness, the individually significant dimensions were affective (*p* = .017) and agentic (*p* < .001). For academic achievement, the individually significant dimensions were affective (*p* = .026) and agentic (*p* < .001).

**Table 6:** SELA as predictor of academic support, competence, relatedness, and academic achievement (Study 2).

	*B* (SE)	β	*p*
Academic support			
Cognitive	.06 (.05)	.05	.258
Affective	.35 (.05)	.32	<.001
Behavioral	.25 (.05)	.25	<.001
Agentic	.16 (.03)	.20	<.001
Competence			
Cognitive	.03 (.05)	.03	.527
Affective	.16 (.05)	.17	.001
Behavioral	.14 (.05)	.17	.002
Agentic	.16 (.03)	.24	<.001
Relatedness			
Cognitive	−.03 (.07)	−.02	.703
Affective	.18 (.07)	.13	.017
Behavioral	.08 (.07)	.06	.252
Agentic	.33 (.05)	.33	<.001
Achievement			
Cognitive	.07 (.05)	.08	.147
Affective	.11 (.05)	.12	.026
Behavioral	.04 (.05)	.05	.363
Agentic	.13 (.03)	.20	<.001

*N* = 404; For Academic support, Adjusted R^2^ = .37, *F* = 59.20, *p* < .001; for Competence, Adjusted R^2^ = .19, *F* = 24.45, *p* < .001; for Relatedness, Adjusted R^2^ = .16, *F* = 19.45, *p* < .001; for Achievement, Adjusted R^2^ = .09, *F* = 11.24, *p* < .001.

### Measurement invariance

To examine whether the same latent model was held in different genders (male *vs*. female) and academic years (1st *vs*. 3rd year), we carried out a group of nested models with indications of equivalence (Table 7). Results showed that HE-SELS presented full configural and metric invariance across genders and academic years. We also verified partial scalar invariance for gender (Δ χ 2 = 24.64, *p* = .006, Δ CFI = .009) and academic year (Δ χ 2 = 24.13, *p* = .004, Δ CFI = .009), after freeing some constraints, two and three, respectively, and only according to the Cheung and Rensvold’s [50] ΔCFI < .01 criterion.

**Table 7:** Invariance: Model comparisons for gender and academic year (Study 2).

	χ ^2^	*df*	χ ^2^/*df*	CFI	Δ χ ^2^	Δ CFI
Invariance for gender						
Configural (factor structure)	220.83	96	2.30	.929		
Metric	235.90	104	2.27	.926	15.07	.003
Scalar	260.53	114	2.29	.917	24.64^**^	.009
Covariances	282.31	121	2.33	.909	21.78^**^	.008
Residuals	306.67	133	2.31	.902	24.36^*^	.007
Invariance for year						
Configural (factor structure)	173.54	96	1.81	.954		
Metric	188.86	104	1.82	.950	15.35	.004
Scalar	213.01	113	1.89	.941	24.13^**^	.009
Covariances	226.87	122	1.86	.938	13.86	.003
Residuals	246.26	134	1.84	.934	19.39	.004

* *p* < .05; ** *p* < .01; *** *p* < .001

Additionally, we verified full residual invariance for the academic year and partial covariance invariance after lifting one additional constraint. As for gender, we obtained full residual invariance only according to Cheung and Rensvold’s [50] ΔCFI criterion, and partial covariance invariance, also only according to Cheung and Rensvold’s [50] ΔCFI < .01 criterion, after freeing three additional constraints. These data suggest measurement invariance, only partial in some levels, for gender and academic years.

## Discussion

### Conceptual clarification

The existing scales to measure SE have several limitations, so this research aimed to develop a robust scale that considers the theories and the conceptual and empirical clarifications suggested by the research. Clarifying the SE concept requires addressing overgeneralizations and object ambiguity. Specifically, the SE concept has become a mixture of indicators, antecedents, and outcomes [6,12] and a blend of SELA and SEAC [13,14,26]. The HESELA-SS clarifies this confusion by focusing only on SELA (not SEAC) and excluding antecedents and outcomes. It defines SE through four dimensions—behavioral, affective, cognitive, and agentic—and provides clear operational indicators. This conceptual framework and the clear delineation of SELA facilitate a more effective analysis of its relationships with student background characteristics (e.g., family variables), facilitators, and outcomes (e.g., academic performance); these can be organized distinctly around SELA, separately from SEAC.

### A psychometrically strong scale

HESELA-SS showed good psychometric qualities, confirming construct validity, internal consistency, test-retest reliability, predictive validity, convergent and discriminant validity, concurrent validity, and measurement invariance. This was likely made possible by the clear conceptual framework that guided HESELA-SS’s development and validation.

The EFA and CFA supported the four-dimensional model (construct validity)—including the agency dimension, as most of the factor loadings were excellent, and there were no cross-loadings [44]. Each 3-item HESELA-SS scale showed good internal consistency. Therefore, the short-scale format with three items per dimension did not compromise the scale’s reliability. However, it is more difficult to obtain adequate reliability values in this type of brief measure [45].

The four HESELA-SS dimensions also showed convergent and discriminant validity, with only the cognitive dimension presenting lower but acceptable values. However, in Study 3, with a different sample, results further supported convergent and discriminant validity.

The HESELA-SS showed concurrent solid validity with the SEQ, which was true for the overall and individual dimension scores. Regarding measurement invariance, scores on the HESELA-SS presented full configural and metric invariance and partial scalar invariance across different genders and academic years. For predictive validity, the agentic and affective dimensions were significant independent predictors of all three criterion variables: competence, relatedness, and academic achievement. These findings align with other research [10,27,52] and suggest that educators and policymakers should place a higher value on the proactive contributions of higher education students in their learning and academic trajectories. Agentic engagement also consistently emerged as the best predictor of a supportive social context [27] and was the best individual predictor of achievement. The addition of this dimension, theoretically framed and psychometrically supported, allows for a broader understanding of how engaged students contribute to their learning. It gains even more importance given that the Bologna declaration [1], put forward by the governments for higher education, which values students’ active participation in their learning process, is the main focus of the agentic dimension [2,16].

### A clear, short, and useful scale

HESELA-SS offers improvements over existing scales regarding item clarity (i.e., a single idea per item), removing any connection to the semantics of the SEAC component and requiring only three items to represent each dimension. With only 12 items, the HESELA-SS offers a psychometrically strong instrument that is also convenient and easy to use because of its short response time. The length of the scale is advantageous in conducting longitudinal studies when researchers ask participants to complete the engagement scale on multiple occasions and in extensive research projects that assess many variables related to SELA [e.g., 34, 35]. Scores can be used to evaluate the four dimensions separately or as an overall total score, as we found no significant difference between the four-factor and the second-order models.

The robust properties of the HESELA-SS make it useful to higher education professors and policymakers seeking information concerning students’ learning and academic experiences. Such information can support prevention and intervention programs by planning quasi-experimental studies with pre- and post-application for group or large-scale assessments. Notably and in line with previous studies [30], HESELA-SS scores have been identified as having significant relations with academic support, underscoring the importance of helpful pedagogical practices in fostering SE and learning outcomes in higher education [8,9,20].

### Theoretical and practical implications

From a theoretical perspective, HESELA-SS enhances our understanding of SE in higher education by clearly defining the concept and using a reliable measurement tool. Our results confirm the value of the four-dimensional model proposed by others [32] and its replicability in different samples. By concentrating on learning, HESELA-SS establishes a comprehensive framework that captures the intricate nature of SE. This clarity aids in distinguishing SE from related concepts.

From the applied point of view, the HESELA-SS can be a practical tool for educators and researchers. With only 12 items, it allows lecturers/teachers to quickly assess student engagement and adjust their practices accordingly. Notably, including agentic engagement highlights students’ proactive roles in their learning. Furthermore, it is an instrument that can be used to identify which type of engagement (cognitive, affective, behavioral, and agentic) needs intervention.

The scale’s robust psychometric properties ensure a reliable assessment of SE and its effects on academic achievement and related outcomes. HESELA-SS can be used to identify and address the major problems of higher education institutions, such as accessibility, quality of education, mental health, and university-to-work transition [2,16]. Firstly, it can help identify disparities in SE, guiding policies aimed at improving accessibility and reducing socio-economic barriers. Secondly, because the university environment strongly predicts SE [5], universities can modify curricula and teaching methods by understanding engagement levels to better align with students. Lastly, it can assist in identifying students facing mental well-being challenges [8,53].

### Limitations and future research

The findings of this study should be considered alongside certain limitations. HESELA-SS could benefit from specifying the incidence of SELA, as a student can be highly engaged in some curricular units but not as engaged in others. Thus, it would be interesting for HESELA-SS to be validated more situationally, focusing on specific contents. Future research could bring additional advances based on a pluri-component structure of the SELA concept.

Additionally, while the HESELA-SS showed solid predictive validity, we measured academic achievement only by students’ self-report ratings. Future studies using institutional ratings are recommended. Moreover, these studies employed a cross-sectional, correlational research design, which prevents any inference regarding causal relationships. Future research on engagement should adopt longitudinal or quasi-experimental designs to investigate the potential causal link between SELA and academic achievement. Finally, cross-cultural studies are suggested to validate the HESELA-SS for other cultures and countries. Portugal’s cultural context is characterized by collectivist values [54]; therefore, results could differ from those of other cultural environments.

## Conclusion

Theory, research, and assessment in the empirical analysis of SE have been shrouded in conceptual haziness and confusion. The present study sought greater conceptual and assessment clarity by overcoming tendencies toward overgeneralization and object ambiguity. The result was a short and four-dimensional scale with robust psychometric properties. With only 12 items, the HESELA-SS scale is a practical and accessible tool for educators and researchers seeking to better understand how higher education students effectively engage in learning activities.

## Supporting information

S1 Fig Items of the HESELA-SS Validated in CFA: Second-order Model (Study 2). (TIF)

S1 Table SELA Scales for Higher Education – Some of the Most Common. (PDF)

S2 Table Forty Candidate Items Developed for the EFA (in Study 1). (PDF)

S3 Table HESELA-SS. (PDF)

S1 File Higher Education Student Engagement in Learning Activities: A Short-Scale (HESELA-SS). (PDF)

S2 File Approval ethics comittee. (PDF)
