# Supplementary figures and images for: Higher education student engagement in learning activities: Clarifying concepts and introducing a short-scale
Source: PLoS One. 2026 Feb 19;21(2):e0340391. doi: 10.1371/journal.pone.0340391 (PMC12919811; doi:10.1371/journal.pone.0340391)

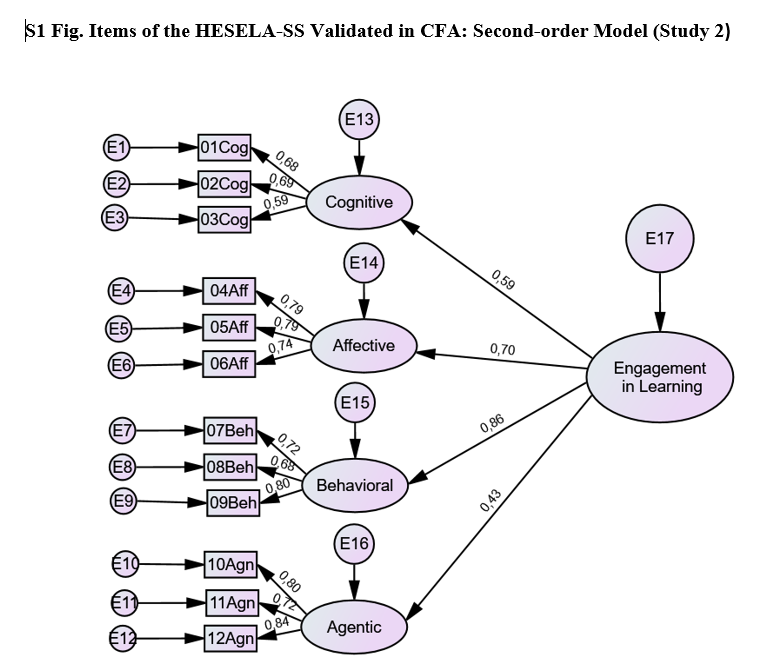

Supplement: S1 Fig — (TIF) [file pone.0340391.s001.tif]
