# Supplementary material for: Higher education student engagement in learning activities: Clarifying concepts and introducing a short-scale
Source: PLoS One. 2026 Feb 19;21(2):e0340391. doi: 10.1371/journal.pone.0340391 (PMC12919811; doi:10.1371/journal.pone.0340391)
Supplement: S1 Table — (PDF) [file pone.0340391.s002.pdf]

S1 Table. SELA Scales for Higher Education – Some of the Most Common

Name of scale	Reference Author(s) (year)	Dimensions	Target group	No. Items	Strengths α	Weakness
National Survey of Student Engagement (NSSE)	[3] Kuh (2001)	Academic challenge, learning with peers, experience with faculty, campus environment	First to fourth-year students in colleges and universities	100 or more, according to the year	Reliability and validity<br>α =.70 to .85	- Survey length: Many items may lead to survey fatigue.<br>- Includes items that are facilitators of engagement<br>- Includes both SEL and SEAC items
Student Engagement Scale (SES)	[13] Gunuc & Kuzu (2015)	Valuing, sense of belonging, cognitive engagement, peer relationships, relationships with faculty members, and behavioral engagement	First to fourth-year university education students	41	Reliability and validity<br>α = 72 to 90	- Includes both SEL and SEAC items
Higher Education Student Engagement Scale (HESES)	[14] Zhoc et al. (2019)	Academic engagement, cognitive engagement, social engagement with peers, social engagement with teachers, and affective engagement	First-year students in higher education	28	Reliability and validity<br>α =.70 to .87	Includes both SEL and SEAC items
The University Student Engagement Inventory (USEI)	[33] Maroco et al. (2016)	Cognitive, emotional, and behavioral dimensions	First to fourth-year university students	15	Reliability and validity; validated for several countries.<br>α = .74 to .89	Not all items are directly focused on learning.

Student Engagement Scale (StES)	[26] Tadesse et al. (2018)	Integrative and collaborative learning, academic challenge, student-teacher interaction, class interactions, assessment tasks, supportive campus environment, enriching learning experiences, interpersonal relationships, and reading and writing	First to third-year university students	38	Reliability and validity<br>α =.63 to .91	- High number of dimensions<br>- Includes both SEL and SEAC items.
