# Supplementary material for: Higher education student engagement in learning activities: Clarifying concepts and introducing a short-scale
Source: PLoS One. 2026 Feb 19;21(2):e0340391. doi: 10.1371/journal.pone.0340391 (PMC12919811; doi:10.1371/journal.pone.0340391)
Supplement: S2 Table — (PDF) [file pone.0340391.s003.pdf]

S2 Table. Forty Candidate Items Developed for the EFA (in Study 1)

Candidate Items for Cognitive Engagement

The following items concern what one thinks during learning.

01. When I study, I try to relate what I am learning with something I already know.
02. When I find a word that I do not know, I try to find out its meaning.
03. When I learn new subjects, I try to associate them with what I have learned in other classes.
04. When I study, I try to see how the information I am learning can be useful to me in real life.
05. I try to integrate subjects from different courses into my general knowledge.
06. I try to create my own examples to help me understand the important concepts to learn.
07. When it is hard to understand a subject, I use different ways of studying it.
08. When I learn new subjects, I try to put them in my own words.
09. I try to integrate my previously learned knowledge to solve new problems.
10. When I study a subject, I try to make the several ideas fit together and make sense.

Candidate Items for Affective Engagement

The following items concern what one feels during learning.

01. When a task comes up in class, I feel very interested in participating.
02. I like what I am learning in class.
03. I feel enthusiastic about what I will learn this academic year.
04. I like to learn new things in class.
05. The classroom is a nice place.
06. I feel that learning gives me satisfaction.
07. I am interested in the schoolwork.
08. I feel that what we are learning in class is very interesting.
09. I feel excited about the work we are doing in class.
10. I like to study at my school faculty or institute.

Candidate Items for Behavioral Engagement

The following items relate to the behavior that occurred during learning.

01. I pay attention in classes.
02. When I start studying, I set aside things that can distract me.
03. The first time the teacher talks about a theme, I listen carefully.
04. I work as much as I can when we start a new subject.
05. I do the scheduled assignments within the set deadlines.
06. I actively participate in the group assignments.
07. During classes, I participate with interest in the tasks that are being carried out.
08. I remain very attentive to the professor's lecture.
09. I read carefully the texts recommended for the classes.
10. I make the most of my studying time.

Candidate Items for Agentic Engagement

The following items concern one’s classroom input and initiative.

01. During class, I express my opinions to the teachers.
02. I let the teachers know what my favourite themes are.
03. I give the professors suggestions to create innovative tasks.
04. During class, I ask questions about the content to learn.
05. I tell the teachers when some subject seems important to me.
06. I speak to the teachers about choosing the assignments in the course.
07. I speak to the teachers about alternative ways to carry out the tasks.
08. I give the teachers suggestions to discuss themes not in the course contents.
09. I let the teachers know what I think about the subjects to learn.
10. I usually look for a good discussion with the teachers to deepen my learning.
