# Supplementary material for: Higher education student engagement in learning activities: Clarifying concepts and introducing a short-scale
Source: PLoS One. 2026 Feb 19;21(2):e0340391. doi: 10.1371/journal.pone.0340391 (PMC12919811; doi:10.1371/journal.pone.0340391)
Supplement: S3 Table — (PDF) [file pone.0340391.s004.pdf]

S3 Table. HESELA-SS

Cognitive Engagement
The following items concern what one thinks during learning.
01. When I learn new subjects, I try to associate them with what I have learned in other classes.
02. When I study, I try to relate what I am learning with something I already know.
03. I try to integrate my previously learned knowledge to solve new problems.
Affective Engagement
The following concern what one feels during learning.
04. I like what I am learning in class.
05. I feel enthusiastic about what I am going to learn this academic year.
06. I feel that what we are learning in class is very interesting.
Behavioral Engagement
The following items relate to the behavior that occurred during learning.
07. I remain very attentive to the professor's lecture.
08. I pay attention in classes.
09. I work as much as I can when we start a new subject.
Agentic Engagement
The following items concern one’s classroom input and initiative.
10. I let the professors know what I think about the subjects to learn.
11. During class, I ask questions about the content to learn.
12. I give the professors suggestions to create innovative tasks.
