# Supplementary material for: Higher education student engagement in learning activities: Clarifying concepts and introducing a short-scale
Source: PLoS One. 2026 Feb 19;21(2):e0340391. doi: 10.1371/journal.pone.0340391 (PMC12919811; doi:10.1371/journal.pone.0340391)
Supplement: S1 File — (PDF) [file pone.0340391.s005.pdf]

Higher Education Student Engagement in Learning Activities: A Short-Scale (HESELA-SS)
(The Portuguese version of the scale, in parenthesis)

The following is a set of items regarding student engagement in learning activities. The answers are on a 5-point Likert scale: 1 (strongly disagree), 2 (disagree), 3 (disagree and agree), 4 (agree), and 5 (strongly agree).

Cognitive Engagement
(Envolvimento Cognitivo)
The following items concern what the student thinks during learning activities.
(As frases seguintes referem-se ao que o estudante pensa durante as atividades de aprendizagem).

1. When I learn new subjects, I try to associate them with what I have learned in other classes.
   (Quando aprendo novas matérias, tento associá-las ao que aprendi em outras aulas).
2. When I study, I try to relate what I am learning with something I already know.
   (Quando estudo, tento relacionar o que estou a aprender com algo que já sei).
3. I try to integrate my previously learned knowledge to solve new problems.
   (Procuro integrar os meus conhecimentos previamente aprendidos para resolver novos problemas).

Affective Engagement
(Envolvimento Afetivo)
The following items concern what the student feels during learning activities.
(As frases seguintes têm a ver com o que o estudante sente durante as atividades de aprendizagem).

4. I like what I am learning in class.
   (Gosto do que estou a aprender nas aulas).
5. I feel enthusiastic about what I am going to learn this academic year.
   (Sinto-me entusiasmado com o que vou aprender este ano letivo).
6. I feel that what we are learning in class is very interesting.
   (Sinto que o que estamos a aprender nas aulas é muito interessante).

Behavioral Engagement
(Envolvimento Comportamental)
The following items relate to the student's behavior that occurs during learning activities.
(As frases que seguem referem-se ao comportamento do estudante durante as atividades de aprendizagem).

7. I remain very attentive to what the professors teach.
   (Mantenho-me muito atento ao que o professor ensina).
8. I pay attention in classes.
   (Presto atenção nas aulas).
9. I work as much as I can when we start a new subject.
   (Trabalho o máximo que posso quando começamos a estudar um novo assunto).

Agentic Engagement
(Envolvimento Agêntico)
The following items refer to the student's initiatives or suggestions to the professor during learning activities.
(As frases seguintes referem-se às iniciativas ou sugestões do aluno ao professor durante as atividades de aprendizagem).

10. I let the professors know what I think about the subjects to learn.
    (Digo aos professores o que penso sobre os temas a aprender).
11. During class, I ask questions about the content to learn.
    (Durante as aulas, faço perguntas sobre os conteúdos a estudar).
12. I give the professors suggestions to create innovative tasks.
    (Dou sugestões aos professores para criar tarefas inovadoras).
