# Supplementary material for: Higher education student engagement in learning activities: Clarifying concepts and introducing a short-scale
Source: PLoS One. 2026 Feb 19;21(2):e0340391. doi: 10.1371/journal.pone.0340391 (PMC12919811; doi:10.1371/journal.pone.0340391)
Supplement: S2 File — (PDF) [file pone.0340391.s006.pdf]

INSTITUTO DE
EDUCAÇÃO
ULISBOA

COMISSÃO DE ÉTICA

PARECER

The Ethical Commission of the Institute of Education, University of Lisbon, received a research project for ethical appreciation, in the area of *Curriculum, Teachers Education and Technology,* titled ***Student Engagement: Conceptual Clarification as Base to a New Measures for Different Levels of Education.***

The analysis of this project revealed a very detailed description of its main components both the conceptual framework and the diverse methodological processes. While considering the ethical foundation of the research project, the answers given to the questionnaire suggest that all the ethical requirements will be followed. Among them, the following processes are highlighted: permission regarding the students participation in the study; the participants sensitivities regarding the data collection process; obtaining the participants consensual and free participation; and anonymity and data protection during and after the end of the project.

The Ethical Commission gave permission to this project to the extent it is in full concordance with the Ethical Chart for Research in Education of the Institute of Education, University of Lisbon.

Institute of Education, University of Lisbon

Lisbon, 26 November 2023

Member of Ethical Comission

Maria de Fátima Chorão Sanches

Professor Maria de Fátima Chorão Sanches

INSTITUTO DE EDUCAÇÃO
Universidade de Lisboa
ENTRADA
Data 27 de 11 de 2023
N.º 1525 Proc.º IDOH

Alameda da Universidade, 1649-013 Lisboa Portugal | T. +351 217 943 633
F. +351 217 943 408 | geral@ie.ulisboa.pt | www.ie.ulisboa.pt
